# Colon Cancer-Associated *Fusobacterium nucleatum* May Originate From the Oral Cavity and Reach Colon Tumors via the Circulatory System

**DOI:** 10.3389/fcimb.2020.00400

**Published:** 2020-08-07

**Authors:** Jawad Abed, Naseem Maalouf, Abigail L. Manson, Ashlee M. Earl, Lishay Parhi, Johanna E. M. Emgård, Michael Klutstein, Shay Tayeb, Gideon Almogy, Karine A. Atlan, Stella Chaushu, Eran Israeli, Ofer Mandelboim, Wendy S. Garrett, Gilad Bachrach

**Affiliations:** ^1^The Institute of Dental Sciences, The Hebrew University-Hadassah School of Dental Medicine, Jerusalem, Israel; ^2^Department of Orthodontics, The Hebrew University-Hadassah School of Dental Medicine, Jerusalem, Israel; ^3^Infectious Disease & Microbiome Program, Broad Institute of MIT and Harvard, Cambridge, MA, United States; ^4^Department of Biotechnology, Hadassah Academic College, Jerusalem, Israel; ^5^Department of General Surgery, Hadassah-Hebrew University Medical Center, Jerusalem, Israel; ^6^Department of Pathology, Hadassah-Hebrew University Medical Center, Jerusalem, Israel; ^7^Department of Gastroenterology, Hadassah-Hebrew University Medical Center, Jerusalem, Israel; ^8^Department of Immunology and Cancer Research, Institute for Medical Research Israel Canada (IMRIC), Hebrew University-Hadassah Medical School, Jerusalem, Israel; ^9^Department of Immunology and Infectious Diseases and Molecular Metabolism, Harvard T. H. Chan School of Public Health, Boston, MA, United States; ^10^Department of Medical Oncology, Dana-Farber Cancer Institute, Boston, MA, United States

**Keywords:** CRC (colorectal cancer), *Fusobacterium nucleatum*, transient bacteremia, oral microbes, cancer

## Abstract

*Fusobacterium nucleatum* is a common oral bacterium that is enriched in colorectal adenomas and adenocarcinomas (CRC). In humans, high fusobacterial CRC abundance is associated with chemoresistance and poor prognosis. In animal models, fusobacteria accelerate CRC progression. Targeting *F. nucleatum* may reduce fusobacteria cancer progression and therefore determining the origin of CRC *F. nucleatum* and the route by which it reaches colon tumors is of biologic and therapeutic importance. Arbitrarily primed PCR performed previously on matched same-patients CRC and saliva *F. nucleatum* isolates, suggested that CRC *F. nucleatum* may originate from the oral cavity. However, the origin of CRC fusobacteria as well as the route of their arrival to the tumor have not been well-established. Herein, we performed and analyzed whole genome sequencing of paired, same-patient oral, and CRC *F. nucleatum* isolates and confirmed that CRC-fusobacteria originate from the oral microbial reservoir. Oral fusobacteria may translocate to CRC by descending via the digestive tract or using the hematogenous route during frequent transient bacteremia caused by chewing, daily hygiene activities, or dental procedures. Using the orthotropic CT26 mouse model we previously showed that IV injected *F. nucleatum* colonize CRC. Here, we compared CRC colonization by gavage vs. intravenous inoculated *F. nucleatum* in the MC38 and CT26 mouse orthotropic CRC models. Under the tested conditions, hematogenous fusobacteria were more successful in CRC colonization than gavaged ones. Our results therefore provide evidence that the hematogenous route may be the preferred way by which oral fusobacteria reach colon tumors.

## Introduction

*Fusobacterium nucleatum* is a Gram-negative, non-spore forming oral anaerobe and one of the abundant species found in the oral cavity (Socransky et al., 1998; Nozawa et al., 2020). *F. nucleatum* is also among the pathobionts that outgrow during dysbiosis preceding periodontal disease (Socransky et al., 1998; Nozawa et al., 2020) to assist keystone species such as *Porphyromonas gingivalis* (Hajishengallis et al., 2011) to drive the breakdown of host-microbial homeostasis and induce periodontitis (Hajishengallis and Lamont, 2016; Lamont et al., 2018).

Genomic studies furnished the earliest evidence that *F. nucleatum* is prevalent in CRC (Castellarin et al., 2012; Kostic et al., 2012). These were followed by reports indicating that *F. nucleatum* plays a role in CRC development (Castellarin et al., 2012; Kostic et al., 2012, 2013; Bullman et al., 2017), metastasis (Li et al., 2016; Bullman et al., 2017; Casasanta et al., 2020; Chen et al., 2020), and disease outcome (Mima et al., 2016; Yu et al., 2017; Brennan and Garrett, 2018). Mechanisms by which *F. nucleatum* promotes tumor progression include generating a proinflammatory tumor-promoting microenvironment (Kostic et al., 2013) and accelerating proliferation of colon cancer cells through activation of Wnt/beta-catenin signaling (Rubinstein et al., 2013, 2019; Wu et al., 2018b) and through TLR4-activated signaling to NF-κB (Yang et al., 2017; Wu et al., 2018b). *F. nucleatum* also supports tumor development by hampering anti-tumor immunity via mechanisms that include interfering with recruitment of tumor infiltrating lymphocytes (Mima et al., 2015; Chen et al., 2018; Hamada et al., 2018) and activating TIGIT and CEACAM1 immune checkpoints that inhibit killing of cancer cells by Natural Killer (NK) cells and tumor infiltrating T cells (Gur et al., 2015, 2019). *F. nucleatum* also induces resistance to chemotherapy in colon cancer via TLR4/NF-κB pathway-induced autophagy (Yu et al., 2017; Zhang et al., 2019). Collectively, these CRC-related fusobacterial effects provide a rationale for the observation that high numbers of *F. nucleatum* in CRC tissue is inversely-correlated with overall survival (Mima et al., 2016) [For review see (Brennan and Garrett, 2018)].

These observations that *F. nucleatum* colonizes CRC, promotes tumorigenesis and affects treatment outcome strongly support that strategies to selectively target bacteria (Zheng et al., 2019) and to hinder their pro-tumorigenic factors should be incorporated into colonic adenoma and CRC treatment. Revealing the origin of CRC fusobacteria and the route by which they reach the tumor, might facilitate development of approaches to reduce numbers of CRC fusobacteria. To date, the “biogeographic” origin of CRC fusobacteria remains under-explored. As *F. nucleatum* is a common oral microbe, and in step with current thinking (Han, 2015; Brennan and Garrett, 2018; Komiya et al., 2018) we hypothesized that *F. nucleatum* found in CRC originate from the oral cavity. Trafficking of oral *F. nucleatum* to CRC can occur by descending through the digestive tract. However, as transient bacteremia is frequent during chewing food, daily oral hygiene activities and during dental procedures (Wilson et al., 2008), hematogenous dissemination of oral fusobacteria to colon tumors is also likely. Here we furnish evidence that CRC fusobacteria are likely oral in origin and that CRC colonization by blood-borne *F. nucleatum* is efficient.

## Materials and Methods

### Collection of Clinical Samples

The Hadassah Medical School IRB approved the use of human samples for this study. Informed consent was obtained from all patients. Patients received neomycin and flagyl orally 12 h before resection as well as metronidazole and cefuroxime IV 30 min prior to resection. For *F. nucleatum* isolation from the oral cavity, 1–2 ml of saliva was collected one day before resection or immediately following colonoscopy. Colonic adenocarcinoma samples (100–1,000 mm^3^) were obtained from the pathology department in sterile tubes within 45 min of resection or colonoscopy. Fresh samples were immediately transferred to an anaerobic chamber (Bactron I-II Shellab, USA) with an atmosphere of 85% N_2_, 5% CO_2_, and 10% H_2_ at 37°C and streaked on Columbia agar plates (Oxoid, UK) supplemented with 5% defibrinated sheep blood (Novamed, Israel) and 0.15% crystal violet. Large resection samples were dissected with a scalpel in the anaerobic chamber before plating. Colonies consistent with *F. nucleatum* morphotypes were verified by Gram staining and microscopy followed by PCR as described below. Oral and CRC fusobacterial strains isolated from the same patient that appeared similar in morphology were kept for further examination.

### Bacterial Strains and Growth Conditions

*F. nucleatum* strains used in this study are listed in Table 1. Fusobacterial strains were grown in Wilkins Chalgren broth (Oxoid, UK) or on Columbia agar plates (Oxoid, UK) supplemented with 5% defibrinated sheep blood (Novamed, Israel) in an anaerobic chamber described above.

**Table 1:** *F. nucleatum* strains used in this study.

**Strain**	**Subspecies**	**SRA identifiers**	**References**	**Source**
CTI3	*animalis*	SRX247711, SRX247710	Broad	Cancer
7_1	*animalis*	SRX110629, SRX347203, SRX104485	McGuire et al. (2014)	Inflamed biopsy tissue (Crohn's patient)
CTI1	*animalis*	SRX247713, SRX247712	Broad	Cancer
4_8	*animalis*	AEIB00000000	McGuire et al. (2014)	Healthy biopsy tissue
3_1_33	*animalis*	SRX111030, SRX276687, RX099485	McGuire et al. (2014)	Inflamed biopsy tissue (Crohn's patient)
OT420	*animalis*	SRX102006, SRX077972, SRX102005	Broad	Reference genome for HMP
CRC003	*animalis*	SRX3688950	This study	Colon cancer
ORAL003	*animalis*	SRX3688949	This study	Oral
T2	*animalis*	SRX3688952	This study	Colon cancer
O2	*animalis*	SRX3688951	This study	Oral
JACRC001	*Polymorphum*	SRX4965003	This study	Colon cancer
JAoral001	*Polymorphum*	SRX4965002	This study	Oral
11_3_2	*animalis*	SRX115841, SRX115973	McGuire et al. (2014)	Healthy biopsy tissue
CTI5	*animalis*	SRX276282, SRX276281	Broad	Cancer
SRR5945983	*animalis*	SRR5945983	Loyola University	Urinary microbiota
SRR2093830	*animalis*	SRR2093830	The Genome Institute at Washington Univ.	Human vagina
4_1_13	*vincentii*	SRR388516, SRR388517, SRR034550	McGuire et al. (2014)	Healthy biopsy tissue
CTI7	*vincentii*	SRX276290, SRX276289	Broad	Cancer
CC53	*vincentii*	SRR317085	Castellarin et al. (2012)	Colon cancer
CTI6	*polymorphum*	SRX276288, SRX276287	Broad	Cancer
13_3C	*polymorphum*	SRX334506, SRX334505, SRX334504, SRX334503	Broad	Reference genome for HMP
ORALGAZE	*polymorphum*		This study	Oral
CTI2	*nucleatum*	SRX247714, SRX247715	Broad	Cancer
DRR015879	*nucleatum*	DRR015879	Gifu Univ. School of Medicine, Japan	/
DRR015964	*nucleatum*	DRR015964	Gifu Univ. School of Medicine, Japan	/

### Cell Lines and Tissue Culture

Mouse cells stably transfected with the luciferase (*luc*) gene CT26 (CT26-luc) and MC38 (MC38-luc), and the human colon adenocarcinoma cell line HT29 were cultured according to ATCC guidelines. These cells were kind gifts from Profs. Panet (CT26-luc), Galun (MC38-luc), and Ben-Neriah (HT29).

### Murine CRC Model

All experiments were performed in accordance with the guidelines of our institutions' animal welfare committee. Mouse orthotopic rectal colon tumors were performed as follow. Male 6–7 weeks old C57BL/6 or Balb/C mice were anesthetized with inhaled isoflurane and injected via the rectum (using a 26-gauge syringe) into the mucosa and submucosa with luciferase expressing colon carcinoma cells MC38-luc or CT26-luc cells, respectively (1 × 10^6^ cells in 100 μl PBS). Real-time imaging using a CCD camera was used to validate tumor development. Luciferin 5 mg/ml was injected intraperitoneally 15 min before image capture. A caliper was used to assess the volume of the tumors and inoculations were conducted when tumor volumes reached ~2,500 mm^3^, calculated as a 3 dimension sphere's volume according to the equation: V = 4/3(π*r*3).

IV inoculation of mice was performed with overnight-grown bacteria washed twice in PBS, in a final volume of 100 μl via tail vein injection using a 27-gauge needle. The bacterial loads used in each IV inoculation are indicated in the figure legends. Gavage inoculation was performed with 200 μl containing ~10^10^ bacteria suspended in final concentration of 1% carboxymethyl cellulose sodium salt (CMC; Sigma, St Louis, MO) in PBS using a mouse gavage needle.

### Quantification of Bacteria Using Plating and qPCR

Murine samples were homogenized using sterile 2 ml tubes with 2.8 mm stainless steel beads in a Fastprep (MP Biomedicals, USA) for 60 s with speed of 6 m/s. Homogenates were serially diluted and plated on Columbia agar plates supplemented with 0.15% (final concentration) crystal violet and 5% (final concentration) defibrinated sheep blood. Plates were incubated in an anaerobic chamber at 37°C for 6 days, and colonies were enumerated. DNA extraction from fresh tissues was performed using the DNeasy Blood & Tissue Kit (Qiagen, Germany), samples were incubated at 56°C overnight. A custom TaqMan primer and probe set was used to amplify *F. nucleatum* DNA. The cycle threshold (Ct) values for *F. nucleatum* was normalized to the amount of murine gDNA in each reaction by using a primer and probe set for the reference gene (*Gapdh*). The fold difference (2^−Δ*Ct*^) in *F. nucleatum* abundance in tumor vs. normal tissue was calculated as described before (Castellarin et al., 2012). Each reaction contained 1 ng of DNA and was assayed in triplicate in 20 μL reactions containing 2 × qPCRBIO Lo-ROX Probe Mix as appropriate for individual qPCR StepOne Real-Time PCR system. Reaction conditions were as follows: 2 min at 50°C, 10 min at 95°C, and 40 cycles of 15 s at 95°C and 1 min at 60°C. The primers and probe sequences for each assay were as follows: *F. nucleatum nusG* forward primer, 5′ ATTGACTTTACTGAGGGAGATTATGTAAAAATC 3′; Fusobacteria FAM probe, 5′-/56-FAM/TCAGCAACT/ZEN/TGTCCTTCTTGATCTTTAAATGAACC/3IABkFQ/-3′. Mouse *gapdh* forward primer, 5′-AATGGTGAAGGTCGGTGTG-3′; Mouse *gapdh* reverse primer, 5′-GTGGAGTCATACTGGAACATGTAG-3′; Mouse *gapdh* FAM probe, 5′-/56-FAM/TGCAAATGG/ZEN/CAGCCCTGGTG/3IABkFQ/-3′.

### Immunofluorescence

MC38 and control HT-29 CRC cells were cultured in 48 well plates overnight. The cells were fixated for 15 min using 4% paraformaldehyde, blocked with PBS supplemented with 10% BSA, 10% FBS, and 0.5% Triton X-100 for 2 h at room temperature and stained overnight at 4°C with FITC-labeled PNA (50 μg/ml in PBS, Sigma-Aldrich). The cells were then incubated with Hoechst 33258 (Sigma-Aldrich) diluted 1:5,000 for 15 min at room temperature washed three times with PBS followed by mounting using Fluoromount aqueous mounting medium (Sigma-Aldrich). Pictures were obtained using the Nikon Eclipse Ti microscope.

### Hemagglutination Assay

Fusobacterial strains were grown overnight, washed twice in PBS, and brought to an OD600 of 1 (~10^9^ CFU ml^−1^). Sheep erythrocytes (RBCs) were washed twice in PBS and brought to a concentration of 2% (vol/vol). 50 μl of fusobacterial cells was mixed with 50 μl of sheep erythrocytes (2% in PBS) in round-bottom 96-well plates (Nunc, Denmark) and incubated at room temperature for 2 h. For inhibition assays, washed bacteria were pre-incubated with 25 mM GalNAc (Sigma-Aldrich) for 30 min prior to incubation with erythrocytes (Coppenhagen-Glazer et al., 2015).

### Genomic Sequencing and Bioinformatics Analysis

Libraries of extracted gDNA were created using the Nextera® XT DNA kit (Illumina, San Diego, USA) according to manufacturer's instructions and sequenced on the Illumina MiSeq platform (Illumina, San Diego, USA) Individually tagged libraries were sequenced in a common flow cell as 2 × 150 base paired-end reads using the Illumina MiSeq platform (Illumina, San Diego, USA).

The gDNA sequence data generated from T2, O2, Oral003, CRC003, JACRC001, and JAoral001 (Table 1) can be accessed under Bioproject PRJNA362799.

### Comparative Analyses

Illumina reads for all *F. nucleatum* genomes sequenced with Illumina technology and deposited at the Sequence Read Archive were downloaded, for a total 18 additional *F. nucleatum* genomes (Table 1). Our comparative dataset included 10 *F. nucleatum* subsp. *animalis* strains: 7_1, 4_8, 11_3_2, 3_1_33, OT420, CTI1, CTI3, CTI5, SRR5945983, and SRR2093830; three *F. nucleatum* subsp. *vincentii* strains: 4_1_13, CTI7, and CC53; three *F. nucleatum* subsp. *nucleatum* strains: CTI2, DRR015879, and DRR015964; and 2 *F. nucleatum* subsp. *polymorphum* strains: CTI6 and 13_3C. For each strain, including T2 and O2, reads were aligned to the *F. nucleatum subsp*. animalis 4_8 reference genome (AEIB00000000) using BWA mem version 0.7.12 (Li and Durbin, 2009). Pilon v.1.12 (Walker et al., 2014) was run in variant discovery mode using default settings to generate a list of single nucleotide polymorphisms (SNPs) for each data set. For phylogenetic reconstruction, a nucleotide alignment was built from all positions containing at least one passing SNP; ambiguous calls in other strains at these positions were scored as Ns. A phylogeny was then estimated using RAxML v7.3.0 (Stamatakis, 2006) using a GTR+Gamma substitution model with 1,000 bootstrap replicates, and midpoint-rooted. The RAxML phylogeny was based on 84,537 positions. To calculate the number of SNPs that differed between two strains, we considered only positions in the Pilon output that had high-quality, passing variant calls, as well as good mapping quality (MQ > 10) in both strains.

For each genome, we obtained draft assemblies using the SPAdes assembler (Bankevich et al., 2012). Using these assemblies, we calculated genome-wide Average Nucleotide Identity (ANI) (Goris et al., 2007) using the online sliding-window implementation available at the Kostas lab (http://enve-omics.ce.gatech.edu/ani/index) with a default window size of 1000bp.

### Statistical Analysis

GraphPad Prism software (Version 6.0) and IBM SPSS Statistics for Windows (Version 25, 2017, Armonk, NY: IBM Corp) were used for statistical presentation and analysis as described in the figure legends.

## Results

### CRC Fusobacteria Originate From Oral Cavity

**Figure 1:**
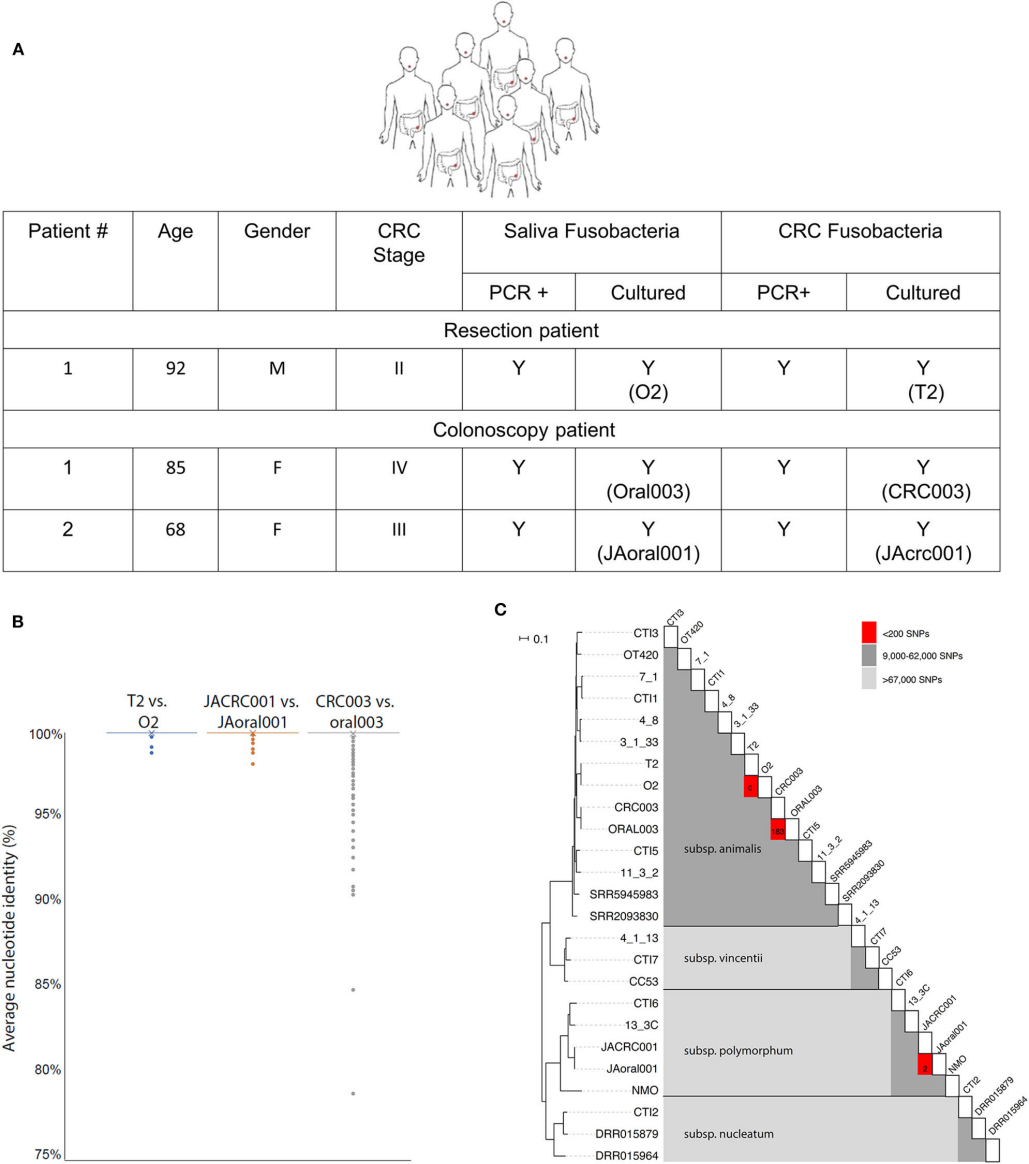
CRC fusobacteria are of oral origin. **(A)** Metadata, saliva, and CRC fusobacteria PCR and culture status of CRC patients. **(B)** The distribution of whole-genome ANI values for each 1,000 bp genomic window for each pairwise comparison of strains. Almost all windows resulted in 100.0% identity, with only a few outliers depicted here (see section Methods). Average ANI values were 100.00 ± 0.03% (T2 vs. O2), 100.00 ± 0.04% (JAoral001 vs. JACRC001), and 99.98 ± 0.38% (CRC003 vs. oral003). **(C)** Midpoint-rooted, SNP-based phylogeny of *F. nucleatum* strains reveals that T2 and O2 are highly related. A heat map illustrating the SNP matrix is shown. For the closely-related strains, the count of SNPs differing is shown in the red boxes. T2 and O2 differ by zero SNPs, and CRC003/ORAL003 differ by 183 SNPs and JAcrc001/JAoral001 differ by 2 SNPs.

To investigate the possible oral origin of CRC—colonized *F. nucleatum*, saliva was obtained from seven patients prior to CRC surgical resection. A matching colon adenocarcinoma sample was obtained from each patient tumor immediately following resection. Fusobacteria could be isolated from all saliva samples, and fusobacterial gDNA was detected by PCR in all tested CRC samples. However, live fusobacteria were only cultured from one colon adenocarcinoma sample (Figure 1A). This CRC isolate, *F. nucleatum* T2, and the paired oral isolate, *F. nucleatum* O2, were biobanked for sequencing and experimental analyses.

Attempts to culture fusobacteria from CRC samples from an additional 23 fusobacteria PCR+ patients were unsuccessful. We hypothesized that the antibiotics that patients received pre-operatively resulted in the low post-operative isolation rate of live fusobacteria. In order to isolate CRC fusobacteria from patients that would not be pre-treated with antibiotics, we identified two patients with high clinical suspicion for CRC who were scheduled for a colonoscopy-based biopsy. CRC tumor samples were collected from these two patients undergoing colonoscopy, who as a matter of practice do not receive pre-procedural antibiotics. Many fusobacterial colonies were obtained from the colonoscopy biopsy samples and from the paired saliva samples. *F. nucleatum* isolates Oral003 and JAoral001 were isolated from these patients' saliva and CRC003 and JAcrc001 were isolated from their CRC samples, respectively (Figure 1A).

Genomic DNA samples from the paired oral and adenocarcinoma fusobacteria were extracted, sequenced and compared to each other and to the fusobacterial genomes previously sequenced using Illumina technology and deposited in the Sequence Read Archive (SRA) database. Two of CRC isolates (T2 and CRC003) belonged to *F. nucleatum subsp. animalis* and the third (JAcrc001) to *F. nucleatum subsp. polymorphum*. Both *F. nucleatum* subspecies *animalis* and *polymorphum* are commonly associated with the oral cavity (Henne et al., 2018). Whole-genome Average Nucleotide Identity (ANI) (Goris et al., 2007) comparisons of the genomes of each isolated oral/CRC pair revealed very high homology with percentage identity for each pair as follows: O2/T2 100.00 ± 0.03%, Oral003/CRC003 99.98 ± 0.38%, and JAoral001/JACRC001 100.00 ± 0.04% (Figure 1B). The paired-patient isolates O2/T2, Oral003/CRC003, and JAoral001/JAcrc001 shared much greater genome sequence identity with one another than they did with other previously sequenced *Fusobacterium* genomes (Figure 1C). Of the reference genome positions where high-quality read mapping was obtained for both T2 and O2 (82% of the *F. nucleatum* subsp. 4_8 reference genome or 1,842,979 positions), there were no single nucleotide polymorphisms (SNPs) separating T2 and O2. Of the reference genome positions where high-quality read mapping was obtained for both Oral003 and CRC003 (82% of the *F. nucleatum* subsp. 4_8 reference genome, or 1,857,684 positions), Oral003 and CRC003 were separated by only 183 SNPs. Of the reference genome positions where high-quality read mapping was obtained for both JAoral001 and JAcrc001 (61% of the *F. nucleatum* subsp. 4_8 reference genome, or 1,371,204 positions), JAoral001 and JAcrc001 were separated by only 2 SNPs. This comparison was performed over a smaller fraction of the reference genome because JAoral001 and JAcrc001 are members of subspecies *polymorphum*, not *animalis*. In comparison, the next most closely homologous fusobacterial genomes, 7-1 and CTI-1, isolated from inflamed biopsy tissue from a Crohn's patient and a CRC tumor, respectively, were separated by 9,553 SNPs (Figure 1C). Thus, our data reveal an extremely close evolutionary relationship between T2 and O2, CRC003 and Oral003, and between JAcrc001 and JAoral001. These data indicate that the strains from each of the paired samples most likely shared a recent common ancestor within each patient and support the hypothesis that fusobacteria from the oral cavity may seed and become enriched in colorectal cancers. Our results support a previous report that used random primed PCR and found very close homology between *F. nucleatum* isolated from CRC and oral cavity of patients (Komiya et al., 2018).

### Oral Fusobacteria Reach CRC via the Blood Circulation

While our data suggest that CRC fusobacteria may originate from the oral fusobacterial community, the route of their oral to CRC transmission remained to be resolved. Our prior studies indicated that fusobacteria can reach the colon tumors by descending from the oral cavity (Kostic et al., 2013) or through the blood (Abed et al., 2016), perhaps as can occur during frequent gingival injury and bleeding (Ashare et al., 2009). Such hematogenous transfer of oral fusobacteria to the placenta was demonstrated previously and may explain the high occurrence of this organism in preterm births (Han et al., 2004).

**Figure 2:**
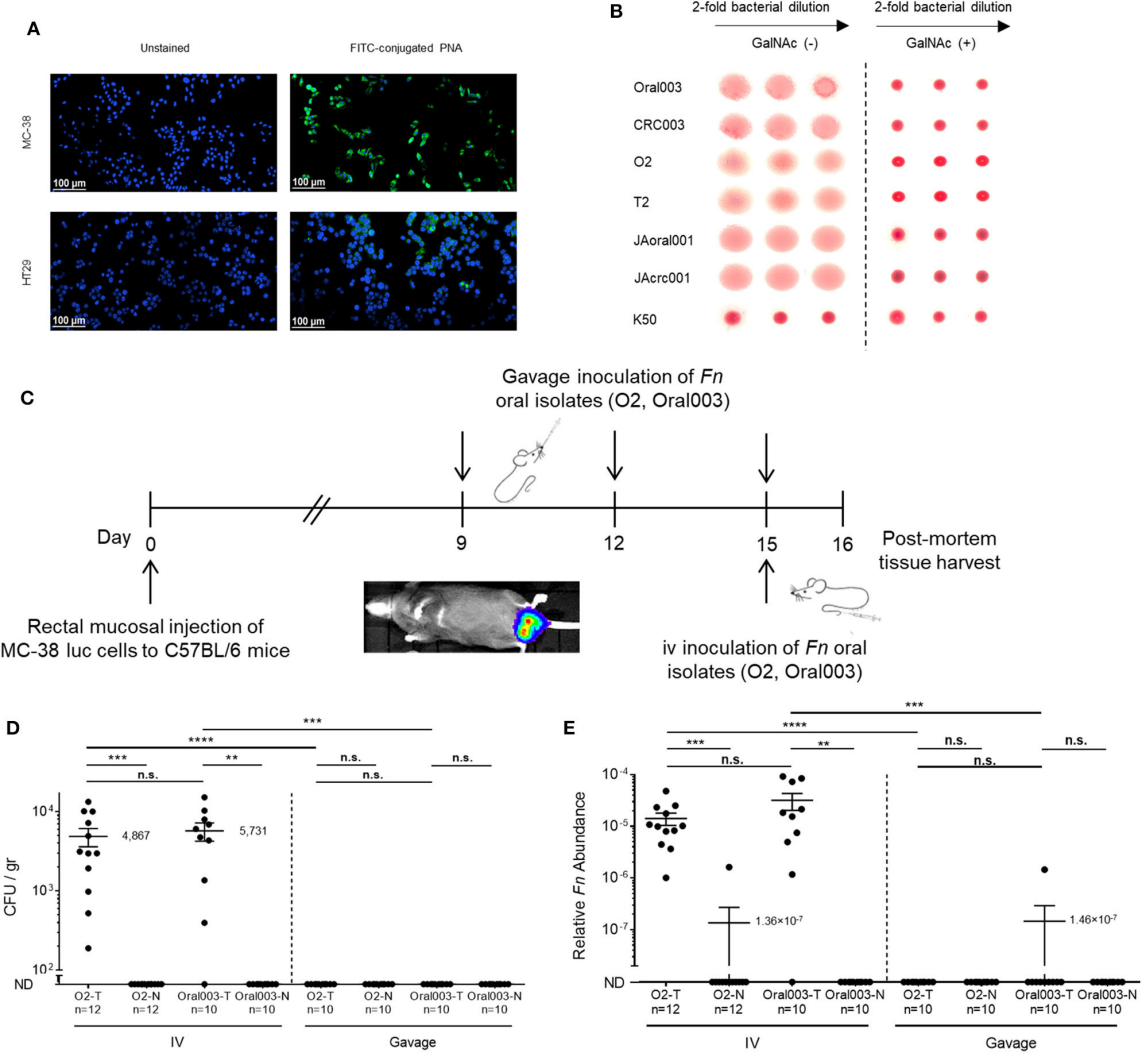
The bloodstream is an efficient route of oral *F. nucleatum* for CRC enrichment. **(A)** MC38 mice CRC cells display high Gal-GalNAc levels. Representative images of MC38 cells and of human adenocarcinoma HT-29 cells unstained (left panels), or stained with FITC-labeled Gal-GalNAc-specific PNA (green, right panels). **(B)** Hemagglutination demonstrating Fap2-dependent Gal-GalNAc binding by matching oral and CRC isolates O2/T2 with Oral003/CRC003 and JAoral001/JAcrc001 in the absence (left) and in the presence (right) of 25 mM GalNAc. The *F. nucleatum* ATCC 23726 Fap2 inactivated mutant K50 was used for negative control. Non-hemagglutinated erythrocytes settle in the bottom of the round bottom well. **(C)** *in vivo* experimental scheme of the orthotopic rectal MC38-luc mouse CRC model. At day 9 post-tumor implantation mice were randomized to an oral or intravenous inoculation group. Oral inoculations were performed on day 9, 12, and 15. A single intravenous inoculation was performed on day 15. **(D,E)** CRC colonization by hematogenously or orally administered fusobacteria. **(D)** Abundance (CFU/gr tissue) and relative fusobacterial gDNA abundance (2^−Δ*Ct*^) **(E)** in tumor (T) samples and in adjacent normal (N) colon samples from MC38 transplanted mice inoculated once with 5 × 10^6^-1 × 10^7^ intravenously (IV) or three times by oral gavage (Gavage) with 10^~10^ *F. nucleatum* O2 or Oral003. ****p < 0.0001, Bonferroni-corrected two-tailed Mann-Whitney test. ***p < 0.001 Bonferroni-corrected two-tailed Mann-Whitney test for gavage vs. IV, Bonferroni-corrected one-tailed Wilcoxon test for tumor vs. normal. **p < 0.01 Bonferroni-corrected one-tailed Wilcoxon test. n.s., not statistically significant. Each symbol represents data from individual mice. Error bars show Mean ± SEM.

To resolve the preferred oral-CRC route, we used the C57BL/6 mouse MC38 colon cancer model. As the intra-rectal MC38 CRC model has not been previously used in studies of fusobacteria, we first confirmed that fusobacteria can bind to MC38 cells. *N*-Acetyl-D-galactosamine (Gal-GalNAc) is displayed at high levels on CRC (Yang and Shamsuddin, 1996) and is correlated with CRC attachment and colonization by fusobacteria (Abed et al., 2016). First, we tested the Gal-GalNAc levels displayed by MC38 cells using FITC-labeled peanut agglutinin (PNA), a Gal-GalNAc [Gal–β(1 → 3)GalNAc] specific lectin and confirmed that MC38 cells display high levels of Gal-GalNAc. We employed human CRC HT29 cells, which display low Gal-GalNAc levels (Abed et al., 2016), as a negative control (Figure 2A). Next, we confirmed the activity of Fap2, the fusobacterial lectin that mediates attachment to CRC-displayed Gal-GalNAc (Abed et al., 2016), in the oral and tumor isolates using standard hemagglutination (Figure 2B). Given these encouraging findings with respect to MC38 cells and the fusobacterial isolates, we proceeded with experiments wherein we implanted MC38 cells stably transfected with the luciferase (luc) gene under the mucosa of the rectum of C57BL/6 wild-type mice. Tumor volume was monitored both by real-time imaging of luciferase expression and by direct measurement of rectal tumors. At day 9 post-tumor implantation, mice were randomized to the oral or the intravascular (IV) inoculation groups and inoculated with two of the new oral *F. nucleatum* isolates O2 or Oral003 according to the experimental scheme presented in Figure 2C. Oral inoculation was performed at day 9 via gavage of ~10^10^ bacteria and repeated two additional times (on days 12 and 15). Intravascular inoculation was performed at day 15, by a single intravenous injection of 5 × 10^6^-1 × 10^7^ fusobacteria via the tail vein. Tumors and adjacent non-cancerous colon samples were harvested 24 h after the last oral or single intravenous inoculation, and fusobacteria were enumerated by plating and by qPCR.

**Figure 3:**
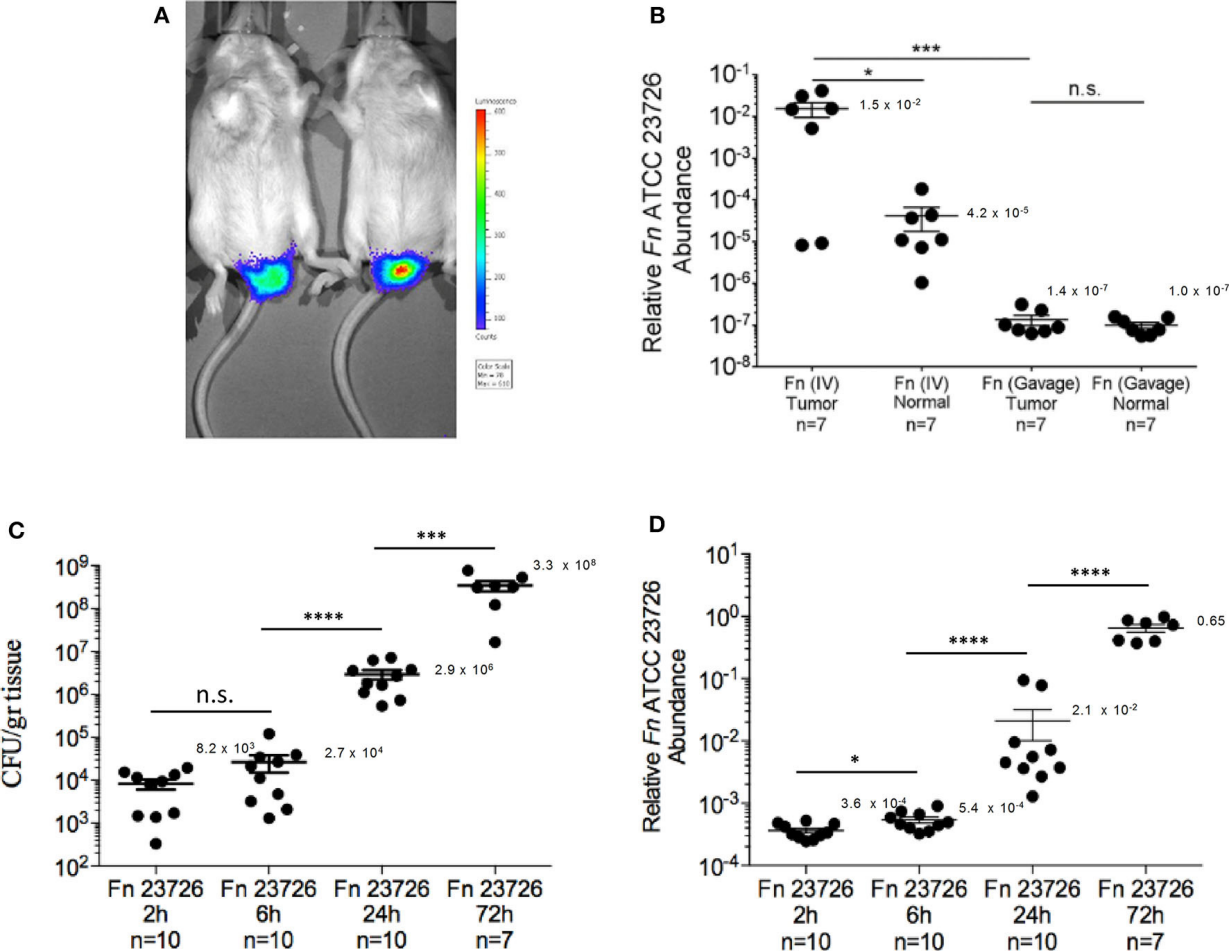
Kinetics of *F. nucleatum* ATCC 23726 enrichment in the CT-26 orthotopic model. **(A)** Detection of implanted CT-26 cells stably transfected with the luciferase (luc) gene under the mucosa of the distal rectum of C57BL/6 wild-type mice. **(B)** Relative fusobacterial gDNA abundance (2^−Δ*Ct*^) in tumor samples and in adjacent normal colon samples from CT26 transplanted BALB/cJ mice inoculated once with 5 × 10^7^-1 × 10^8^ intravenously (IV) or three times by oral gavage (Gavage) with 10^~10^ *F. nucleatum* ATCC 23726. *p < 0.05, Bonferroni-corrected two-tallied Wilcoxon test, ***p < 0.001, Bonferroni-corrected two-tailed Mann-Whitney test. **(C)** Abundance (CFU/gr tissue) and **(D)** relative fusobacterial gDNA abundance (2^−Δ*Ct*^) in tumor samples collected 2, 6, 24, and 72 h after IV inoculation of CT26 transplanted BALB/cJ mice with 5 × 10^7^-1 × 10^8^ *F. nucleatum* ATCC 23726. *p = 0.02, ***p = 0.0001, ****p < 0.0001, Bonferroni-corrected two-tailed Mann-Whitney test. Error bars show Mean ± SEM.

CRC colonization with IV inoculation was far more efficient than with gavage of the oral fusobacteria. Fusobacteria could be cultured from the CRC tumors of all 12 mice IV inoculated with O2 (mean 4.9 × 10^3^ CFU/gr tissue) and from 9 of the 10 mice inoculated IV with Oral003 (mean 5.7 × 10^3^ CFU/gr tissue). In contrast, fusobacteria could not be cultured from any mice orally inoculated with O2 (*n* = 10 mice) or with Oral003 (*n* = 10 mice) (Figure 2D). Fusobacterial gDNA was detected and quantified in the CRC tumors of all above 12 mice IV inoculated with O2 and in 9 out of the 10 inoculated with Oral003. In contrast, fusobacterial DNA was below the limit of detection in the CRC tumors of all 10 mice inoculated by oral gavage with O2 and in 9 out of the 10 mice inoculated with Oral003 (Figure 2E). These results were repeated using a different *F. nucleatum* sub species, *F. nucleatum* ATCC 23726 subsp. *nucleatum* and a different mouse model, the CT26-luc colon cancer model in the BALB/cJ mouse (Figure 3A). Once again, the hematogenous route (a single injection of 5 × 10^7^-1 × 10^8^ fusobacteria) appeared much more efficient than the digestive tract for fusobacteria trafficking to CRC orthotopic tumors. Relative fusobacterial gDNA abundance revealed that gavage-inoculated *F. nucleatum* ATCC 23726 was 1.13 × 10^5^ fold less frequent in the colon cancer as compared to intravascular injected ones, as quantified by qPCR (mean 2^−Δ*Ct*^ of 1.356 × 10^−7^ vs. 0.01538, respectively) (Figure 3B). Increasing the dose of the IV injected bacteria 10-fold from 5 × 10^6^-1 × 10^7^ used in the MC38 model to 5 × 10^7^-1 × 10^8^ used in the CT26 model, resulted in higher levels of fusobacteria in the CRC. However, these differences might also result from the different CRC models and fusobacterial strains used. Intravenously–inoculated fusobacteria were found in mouse CT26 CRC tumors 2 h post-delivery and levels remained stable at 6 h post-infection. This was followed by fusobacterial proliferation in the tumor that continued 24 and 72 h post-infection (Figures 3C,D).

**Figure 4:**
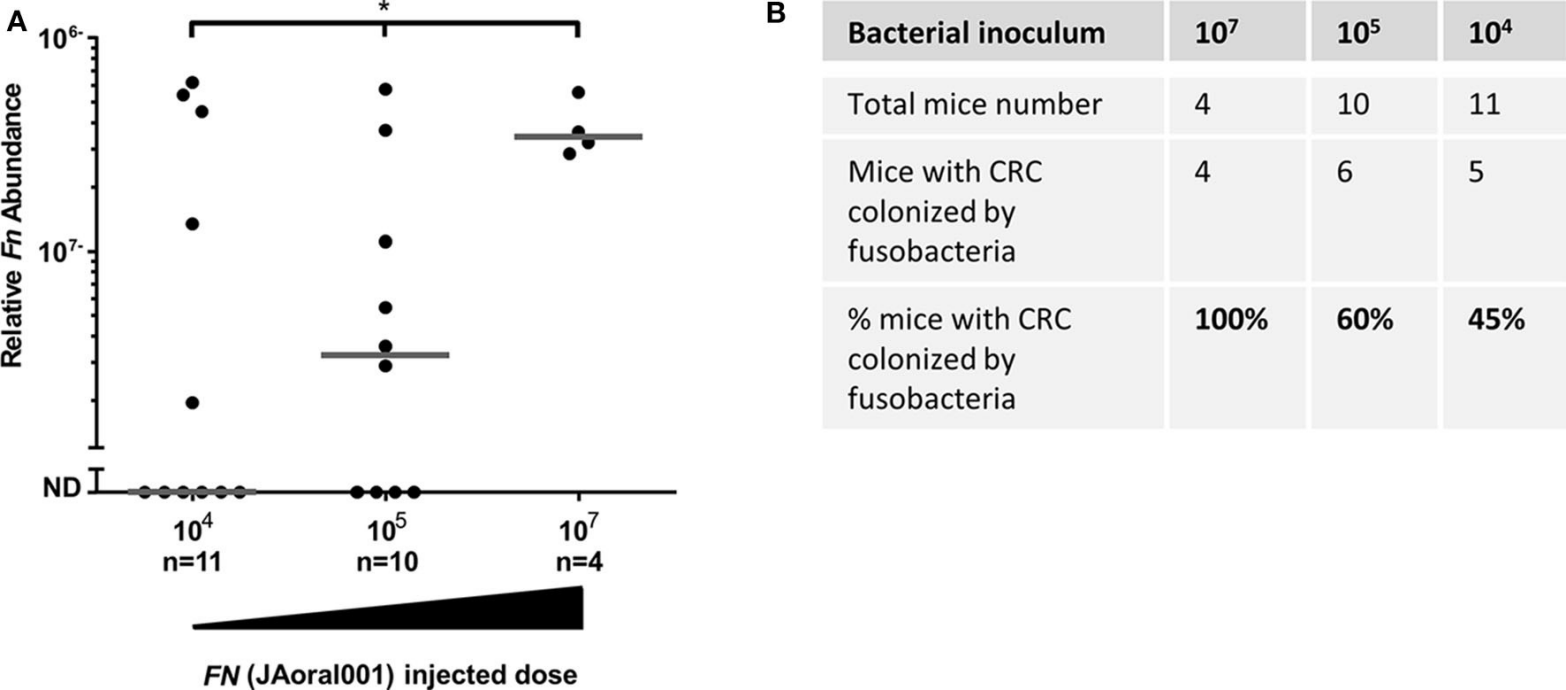
Efficiency of CRC colonization by hematogenous fusobacteria is dose-dependent. **(A)** Relative fusobacterial gDNA abundance (2^−Δ*Ct*^), and proportion CRC colonization **(B)** in tumor samples from MC38 transplanted mice intravenously inoculated daily three times with 5 × 10^3^-1 × 10^4^, 5 × 10^4^-1 × 10^5^ or with 5 × 10^6^-1 × 10^7^ *F. nucleatum* JAoral001. Each symbol represents one mouse. Bar show median. *p < 0.05, the proportion of mice with fusobacterial-colonized tumors increases significantly with the amount of bacteria injected, as determined by an exact one-sided Permutation Test implemented by StatXact* (*p* = 0.0442 with the scores set equal to the amount of bacteria, *p* = 0.04633 with the scores equal to the log).

The magnitude of bacteremia resulting from a dental procedure and from routine daily activities is significantly lower (< 10^4^ CFU/ml) (Wilson et al., 2008) than that tested in our experiments. We therefore tested whether fusobacteria inoculated in doses mimicking these physiological ones (5 × 10^3^-1 × 10^7^) would be found in CRC tumors. We employed the orthotropic MC38 CRC model and *F. nucleatum* JAoral001 in these experiments. CRC tumor-associated fusobacteria could be detected in mice inoculated across the more physiologic dose range. Increasing the dose resulted in increases in the proportion of mice with fusobacteria detected in the tumors. Fusobacteria were found in the tumors of 45% of mice inoculated with 5 × 10^3^-1 × 10^4^, 60% inoculated with 5 × 10^4^-1 × 10^5^, and 100% inoculated 5 × 10^6^-1 × 10^7^ (Figures 4A,B). These results may explain the heterogeneity observed in fusobacterial occurrence and levels in human CRC.

## Discussion

Our results, admittedly in a limited subset of patients, support the concept that fusobacteria, which are abundant in CRC and play a role in prognosis and in treatment outcome, may originate from the oral cavity. In a pre-clinical model, the circulatory system appears to be the most efficient route by which fusobacteria reach colon tumors. In humans, this most likely occurs during transient physiologic bacteremias originating from the mouth.

There are many diseases associated with oral bacteria disseminating to a distant site as a result of transient bacteremia and the paragon is infective endocarditis (IE). Oral *viridans* group streptococci are implicated as causal organisms in 35–45% of cases (Dayer et al., 2015). Administration of antibiotic prophylaxis before invasive dental procedures for prevention of IE has been practiced for over 50 years and remains the global standard of care for patients at risk (Dayer et al., 2015). Transient bacteremia is common with manipulation of the teeth and periodontal tissues. However, there is a wide variation in the reported frequencies of bacteremia in patients resulting from dental procedures: tooth extraction (10–100%), periodontal surgery (36–88%), scaling and root planing (8–80%), and teeth cleaning (up to 40%) [For review see Wilson et al. (2008)]. Transient bacteremias also occurs during frequent daily activities such as tooth brushing and flossing (20–68%) and chewing food (7–51%) (Wilson et al., 2008). Thus, the frequency of bacteremia from routine daily activities is far greater than that from dental treatment.

In our experimental data shown in Figure 4B, lowering the fusobacterial inoculation dose did not abolish CRC colonization but rather reduced its efficiency (measured as frequency of CRC-fusobacteria colonized mice) in a dose-dependent manner. A large body of evidence suggests that the abundance of oral bacteria in the blood during transient bacteremia is approximately 10^4^ CFU/ml. Of course, not all oral bacteria are fusobacteria and the average blood volume of an adult 70 kg male is ~5 L.

Resistance to killing by phagocytes in the blood might be a mechanism to advance hematogenous dissemination by bacteria. *F. nucleatum* can modulate neutrophil function to increase tissue damage (Katsuragi et al., 2003) and deplete neutrophils at the site of oral infection (Jewett et al., 2000; Wu et al., 2018a). However, a report of microbicidal assays indicated that < 98% of *F. nucleatum* organisms were killed by PMNs within 60 min (Mangan et al., 1989). Complicating the matter further, different strains of *F. nucleatum* impact neutrophil responses differently (Kurgan et al., 2017). Therefore, it remains to be elucidated whether *F. nucleatum* is specifically adapted for survival while in transit in host blood to avoid killing by neutrophils.

We do not know if fusobacteria within the bloodstream travel to the tumor as free bacteria or bound to erythrocytes which fusobacteria are well-known to agglutinate. As fusobacteria are non-motile, it is plausible that the altered vasculature of tumors with enhanced blood vessel permeability may assist fusobacteria in reaching tumors. Unlike other bacteria that may arrive in the vicinity of a tumor in a similar manner, fusobacteria are equipped with the Fap2 lectin that binds Gal-GalNAc, which is specifically and highly displayed on many colon tumors (Abed et al., 2016), enabling fusobacteria to bind, persist, and colonize. Tumor-induced local hypoxia and immune effectors might be additional factors that provide fusobacteria with a competitive advantage in the CRC tumor milieu.

Our observation that the intravenous route for CRC colonization by oral fusobacteria is more efficient than a gastrointestinal route in orthotropic CRC mouse models cannot rule out that oral fusobacteria constantly swallowed in humans may colonize CRC through the digestive tract. However, hematogenous spread of oral fusobacteria to CRC is biologically more plausible, as travel through the bloodstream circumvents the toxicity of low gastric pH as well as bile acids encountered when descending through the gastrointestinal tract. Furthermore, bloodstream travel affords fusobacteria an escape from competition with the endogenous colonic microbiota. Also, genomic studies have not uncovered type III secretion systems as part of the *F. nucleatum* pan-genome that would facilitate epithelial cell invasion (McGuire et al., 2014). Tumor arrival via the circulatory system might also explain fusobacterial absence from biofilms in the colonic mucus layer of adenomas of familial adenomatous polyposis patients and some sporadic CRC patients (Dejea et al., 2018).

*F. nucleatum* is currently classified into *five* subspecies: *F. nucleatum* subsp. *nucleatum, F. nucleatum* subsp. *polymorphum, F. nucleatum* subsp. *fusiforme, F. nucleatum* subsp. *vincentii*, and *F. nucleatum* subsp. *animalis*. On average, human adults carry three *F. nucleatum* sub species in their oral cavity (Henne et al., 2018). Two out of the three CRC *F. nucleatum* isolated by us (T2 and CRC003), belong to *F. nucleatum* subsp. *animalis*. Our results support previous reports that *F. nucleatum* subsp. *animalis* is the most prevalent *F. nucleatum* subspecies in human colorectal cancers (Ye et al., 2017). Interestingly, *F. nucleatum* subsp. *animalis* is not the most prevalent *F. nucleatum* subspecies in the oral cavity (Henne et al., 2018) suggesting that *F. nucleatum subsp*. *animalis* may be better adapted for CRC colonization. A recent study suggested that fusobacteria travel intracellularly with the primary tumor cells to distant sites, as part of metastatic tissue colonization (Bullman et al., 2017). We previously showed that Gal-GalNAc is over displayed in CRC metastases (Abed et al., 2016) that may facilitate fusobacterial attachment via Fap2. Our results here suggest that fusobacteria can also reach metastases from the primary tumor or from the oral cavity via the circulatory system.

In conclusion, it is our hope that understanding the mechanisms by which fusobacteria colonize colorectal cancers and accelerate their development and progression may provide a means to more effectively detect, prevent and treat CRC.

## Data Availability Statement

The datasets presented in this study can be found in online repositories. The names of the repository/repositories and accession number(s) can be found at: https://www.ncbi.nlm.nih.gov/bioproject/?term=PRJNA362799, PRJNA362799.

## Ethics Statement

The animal study was reviewed and approved by the Hebrew University of Jerusalem Ethics Committee. The studies involving human participants were reviewed and approved by Hadassah Medical School IRB. The patients/participants provided their written informed consent to participate in this study.

## Author Contributions

JA and NM designed and carried out experiments and participated in writing the manuscript. AM, AE, LP, JE, MK, ST, and SC carried out experiments and participated in writing the manuscript. GA, KA, and EI collected human samples and participated in writing the manuscript. OM, GB, and WG designed experiments and participated in writing manuscript. All authors contributed to the article and approved the submitted version.

## Conflict of Interest

The authors declare that the research was conducted in the absence of any commercial or financial relationships that could be construed as a potential conflict of interest.
